# Reply to: The pressure gradient for venous return and its derivatives are ambiguous measures

**DOI:** 10.1186/s40635-024-00698-5

**Published:** 2024-12-05

**Authors:** Anders Aneman, Markus Benedikt Skrifvars, Koen Ameloot

**Affiliations:** 1 Intensive Care Unit, Liverpool Hospital, South Western Sydney Local Health District and South Western Sydney Clinical School, University of New South Wales, Sydney, Australia; 2 The Ingham Institute for Applied Medical Research, Sydney, Australia; 3 Faculty of Health Sciences, Macquarie University, Sydney, Australia; 4 Department of Emergency Care and Services, Helsinki University Hospital and University of Helsinki, Helsinki, Finland; 5 Department of Cardiology, Ziekenhuis Oost-Limburg, Genk, Belgium; 6 Departement de Cardiologie/Soins Intensifs Adultes, CHC-Montlégia, Liège, Belgique; 7 Department of Cardiology, University Hospitals Leuven, Louvain, Belgium; 8 Faculty of Medicine and Life Sciences, University Hasselt, Diepenbeek, Belgium

**To the Editor,**

We are grateful to Drs. Kenny and Werner-Moller for their interest in our study and their valuable comments [1]. The authors draw attention to the importance of resistance to venous return (RVR), that in the original publication, introducing the mean systemic pressure analogue (*P*_msa_) was defined as the resistance encountered by the average circulating element in returning to the heart [2]. The RVR is a complex variable influenced by both arterial and venous resistances and the proportioning of circulating elements to the arterial and venous volumes depending on regional compliances. The position of resistive influence(s) along the arteriovenous circuit also matters with changes in capacitance becoming more important the closer they occur to the large conduit-veins. Furthermore, viewing RVR as an ohmic, linear phenomenon is likely overly simplistic given the varying pulsatile nature or flow [3]. We agree with the authors that the assumption of constant RVR may be challenged, and this should be considered in future studies of P_msa_ and its derived variables.

As a corollary to RVR not being constant, the heart efficiency (*E*_h_) and volume efficiency (*E*_vol_) variables are described by Drs. Kenny and Werner-Moller as ambiguous. The concepts illustrated in their geometrical model are important to advance our understanding of venous return physiology. We want to clarify our interpretation of the NEUROPROTECT data for the period highlighted by the authors (10–15 h after admission to ICU). It is important to note that while the separation in venous return driving pressure (VRdP = *P*_msa_ – RAP [right atrial pressure]) appears greater around this point in time based on the marginal means, *E*_h_ for the corresponding period was not statistically different, nor was cardiac output (CO). We have not reported an increase in *E*_h_, and do not agree that the data would suggest an improvement in cardiac function. In Fig. 1, we reproduce the proposed geometrical model with data for 10–15 h compared to 30–36 h when cardiac output was significantly greater, while not different between the study groups. For 10–15 h, the intervention group had a slightly higher RVR, but the similar CO means that the intersections with the cardiac function line are almost identical, suggesting that the operating point of the heart has not changed. In the later period, CO increased with a greater separation of VRdP and the RVR still higher in the treatment group. The intersection points are again almost inseparable between treatment groups, even if a hypothetical improvement in cardiac function is assumed. The changes in VRdP induced by the NEUROPROTECT protocol were relatively minor and with interventions that result in greater changes in *P*_msa_ and VRdP, the advantages of the geometrical model suggested by Drs. Kenny and Werner-Moller might become more apparent.

**Fig. 1:**
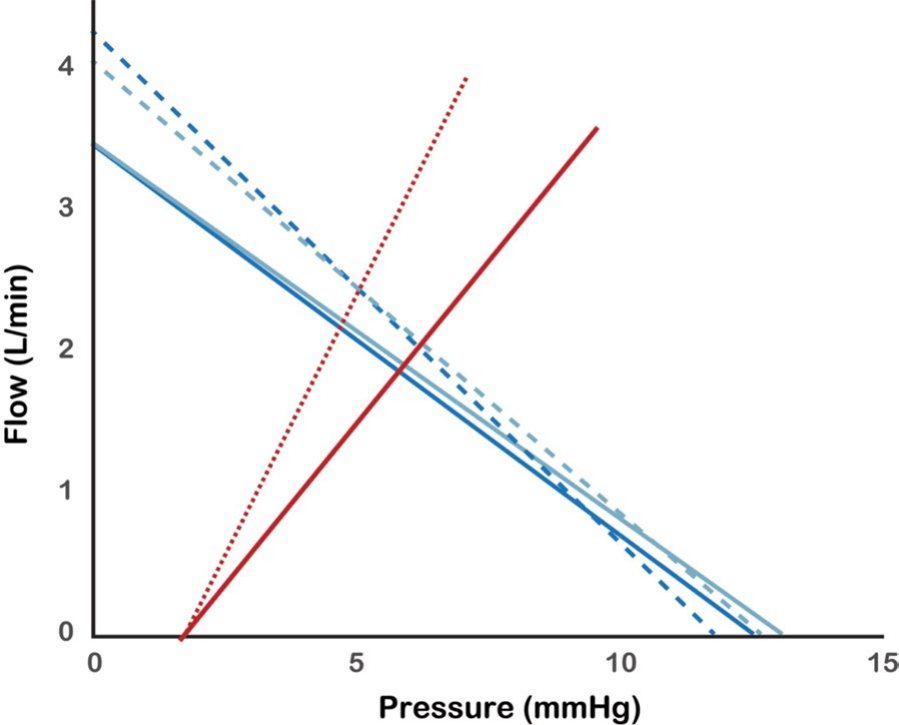
Venous return curves for the control (dark blue lines) and intervention (light blue lines) groups in the NEUROPROTECT trial at 10–15 h (solid lines) and 30–36 h (dashed lines) after admission to ICU. The cardiac function curve added in the geometrical model of Drs. Kenny and Werner-Moller is shown by the solid red line with a hypothetical improvement in cardiac function shown by the red dotted line.

The *E*_vol_ variable essentially represents the slope of the non-linear Frank–Starling curve at an operating point for CO that equates to VRdP. Changes in VRdP are not isolated to the *x*-axis but correlated to changes in flow on the *y*-axis. A degree of mathematical coupling exists between changes in *P*_msa_, VRdP and CO the since calculation of *P*_msa_ incorporates CO. This becomes less of an issue when changes in CO are predicted [4, 5] rather than correlated as in the NEUROPROTECT post-hoc study. While *E*_h_ was not statistically significantly different over time, *E*_vol_ was reported for all bolus episodes and *E*_h_ might have been numerically different. We view the *E*_h_ and *E*_vol_ variables as complementary in the sense that they identify the point and the slope of the intersection between the venous return and cardiac function curves.

In summary, we maintain that the interpretation of an unchanged cardiac function and a greater volume responsiveness in the post-hoc study of the NEUROPROTECT data is correct. We appreciate the valid comments made in the letter by Drs. Kenny and Werner-Moller that warrant exploration in larger datasets encompassing a greater variability in *P*_msa_ and VRdP, with extended consideration given to changes in RVR.

## Data Availability

No data specific to this letter are available.
